# Immediate vs. delayed insertion of intrauterine contraception after second trimester abortion: study protocol for a randomized controlled trial

**DOI:** 10.1186/1745-6215-12-149

**Published:** 2011-06-14

**Authors:** Wendy V Norman, Janusz Kaczorowski, Judith A Soon, Rollin Brant, Stirling Bryan, Konia J Trouton, Lyda Dicus

**Affiliations:** 1 Contraception & Abortion Research Team, Women's Health Research Institute, Vancouver, British Columbia, V6H 1G3, Canada; 2 Department of Family Practice, University of British Columbia, Vancouver, British Columbia, V6T 1Z3, Canada; 3 Faculty of Pharmaceutical Sciences, University of British Columbia, Vancouver, British Columbia, V6T 1Z3, Canada; 4 School of Population and Public Health, University of British Columbia, Vancouver, British Columbia, V6T 1Z3, Canada; 5 Department of Statistics, University of British Columbia, Vancouver, British Columbia, V6H 3V4, Canada; 6 The Centre for Clinical Epidemiology & Evaluation, Vancouver Coastal Health Research Institute, Vancouver, British Columbia, V5Z 1M9, Canada; 7 Vancouver Island Women's Clinic, Victoria, British Columbia, V9B 1T2, Canada; 8 CARE Program, British Columbia Women's Hospital and Health Centre, Vancouver, British Columbia, V6H 3N1, Canada

## Abstract

**Background:**

We describe the rationale and protocol for a randomized controlled trial (RCT) to assess whether intrauterine contraception placed immediately after a second trimester abortion will result in fewer pregnancies than current recommended practice of intended placement at 4 weeks post-abortion. Decision analysis suggests the novel strategy could substantially reduce subsequent unintended pregnancies and abortions. This paper highlights considerations of design, implementation and evaluation of a trial expected to provide rigorous evidence for appropriate insertion timing and health economics of intrauterine contraception after second trimester abortion.

**Methods/Design:**

Consenting women choosing to use intrauterine contraception after abortion for a pregnancy of 12 to 24 weeks will be randomized to insertion timing groups either immediately (experimental intervention) or four weeks (recommended care) post abortion. Primary outcome measure is pregnancy rate at one year. Secondary outcomes include: cumulative pregnancy rates over five year follow-up period, comprehensive health economic analyses comparing immediate and delayed insertion groups, and device retention rates, complication rates (infection, expulsion) and, contraceptive method satisfaction. Web-based Contraception Satisfaction Questionnaires, clinical records and British Columbia linked health databases will be used to assess primary and secondary outcomes. Enrolment at all clinics in the province performing second trimester abortions began in May 2010 and is expected to complete in late 2011. Data on one year outcomes will be available for analysis in 2014.

**Discussion:**

The RCT design combined with access to clinical records at all provincial abortion clinics, and to information in provincial single-payer linked administrative health databases, birth registry and hospital records, offers a unique opportunity to evaluate such an approach by determining pregnancy rate at one through five years among enrolled women. We highlight considerations of design, implementation and evaluation of a trial expected to provide rigorous evidence for appropriate insertion timing and health economics of intrauterine contraception after second trimester abortion.

**Trial registration:**

Current Controlled Trials ISRCTN19506752

## Introduction

Abortion is common in Canada with 96,815 reported in 2005 [1]. Canadian women seeking abortion represent a high risk group for recurrent unintended pregnancy as 38% have had at least one previous abortion [2]. About 12% of all abortions occur past the 12th week in pregnancy (second trimester)[3,4]. Women seeking abortion in the second trimester are disproportionately from marginalized and vulnerable populations [3-6], and recurrent unintended pregnancies may further exacerbate social and economic disadvantages [7]. The most effective contraceptive is "forgettable"[8], that is: a method requiring user attention no more often than every 3 years. Intrauterine contraception (IUC) is efficacious, and thus highly effective contraception [9-12]. Robust evidence exists to favour immediate insertion of an IUC after first trimester abortion [13-19] and post-partum [20-23].

To minimize expulsion and potential for perforation of the IUC device, current recommended care and product monographs indicate delaying insertion after a second trimester abortion until substantial uterine involution (return to non-pregnant size): typically 4-6 weeks [24,25]. This recommendation appears to be founded upon a theoretical risk of greater rates for expulsion prior to uterine involution, as we were unable to find published evidence to support this assertion. Although the rates for both expulsion and perforation are believed to be only marginally higher than for insertion at 4 weeks [26,27], as few as 26% of women return by 6 weeks for a planned delayed insertion [28].

British Columbia (BC) administrative health databases track health care services provided within a single-payer universal health care system. These databases enable a linkage of study participant information to health system data on all births, abortions, miscarriages, any pregnancy related visits, hospital admissions and prescriptions dispensed following study enrollment. This method can substantially reduce attrition rate in a population known to have low post abortion follow up adherence [27-31].

The proposed randomized controlled trial (RCT) of immediate compared to a planned delayed device insertion following second trimester abortion is the first to examine both the levonorgestrel and the copper device and the first to report on pregnancy rate at one year as the primary outcome measure. The study will provide generalizable results using an intention-to-treat framework for the hypothesis: *Intrauterine contraception placed immediately after a second trimester abortion will result in fewer pregnancies than current recommended care of intended placement at 4 weeks post-abortion*. The health economic analysis of costs and cost-effectiveness will facilitate determination of population health implications to inform health systems and health care delivery decisions.

## Methods

### Study design

Women having a second trimester abortion at any BC abortion clinic, and choosing an IUC for post-abortion contraception will be eligible to participate in the study. Consenting participants choose either a copper or a levonorgestrel-releasing IUC and are then randomly allocated to an insertion time immediately or four weeks after their abortion. Contraception Satisfaction Questionnaires (CSQ), clinical records and the linked provincial health administrative databases will be accessed to determine all pregnancies occurring within one year of enrollment, supplemented with a variety of secondary outcomes.

### Sample Size and Power Calculations

350 women choosing a levonorgestrel-releasing IUC (LNG-IUC, "Mirena^®^", Bayer Inc, Canada), and 366 women choosing a "T" shaped copper IUC with 380 mm^2 ^surface area of copper including copper bands on the "T" arms (CuT380-IUC, "Flexi-T380(+)^®^", Prosan, The Netherlands) will be recruited and randomly allocated to each of two treatment arms: immediate insertion (experimental intervention) vs. planned delayed insertion (recommended care), representing a total enrollment of 716 women into this Phase IIIb RCT (See Table 1). This sample size will provide 90% power in the levonorgestrel stratum to distinguish predicted one year pregnancy rates of 1.2% (immediate) versus 8.7% (delayed) and 80% power for corresponding detection of 2.7% and 9.7% in the copper device users.

**Table 1:** Enrollment Allocation

	Immediate	Delayed	Total
LNG-IUC	175	175	350

CuT380-IUC	183	183	366

Total	358	358	716

### Estimation of Probable Rates of Pregnancy

The pregnancy rates used in our sample size calculation are justified as follows. The one year failure rate of the LNG-IUC device is known to be 0.1%[32] except in the case of spontaneous expulsion that we conservatively estimate occurs when immediately inserted after second trimester abortion with a probability of 0.05 (observed rate 0.03 by Drey [27] and Cremer [29]). Our estimated post-abortion pregnancy probability in the absence of an IUC is 0.24 [33]. Therefore the estimated pregnancy rate in the immediate insertion LNG-IUC group is expected to be:

In the delayed group, we conservatively estimate 65% of women will return for insertion of the IUC (Stanek found 26% return for planned delayed insertion [28], and Cremer 30%[29]), and we assume (conservatively) the high expulsion rate of the immediate insertion group. These assumptions imply a one-year pregnancy rate of

The one year failure rate for the CuT380-IUC is 1.7%[34]. Using the above formula this yields rates for immediate and delayed insertion of .028 and .102 respectively. Allowing for a loss to follow-up of up to 5% provides conservative estimates of the observed pregnancy rates of .012 and .0874 in the LNG-IUC cohort and .027 and .097 in the CuT380-IUC group.

### Inclusion Criteria

All women at participating study sites who have completed informed consent for an abortion over 12 and under 24 weeks gestational age (as determined by ultrasound), who are residents of BC enrolled in the universal provincial medical services plan and have chosen to use an IUC for post-abortion contraception are eligible to participate.

### Exclusion Criteria

Women are not eligible if they intend to move from BC within the next year or if they intend to conceive within one year. In addition, if they have a contraindication to the use of the IUC they have chosen (see Table 2) or are currently enrolled in another clinical trial they will be excluded. Post randomization exclusion factors include perforation or excessive bleeding at the time of their abortion or uterine anomaly detected at the time of the abortion procedure. These exclusions are designed to be only those which, in real life, would preclude a woman from being able to choose this method of contraception. This study has no minimum age criteria for enrollment.

**Table 2:** Inclusion and Exclusion Criteria

*Inclusion Criteria - This study will be offered to women at the study sites who meet all of the following criteria: *
Have completed informed consent for an abortion over 12 and under 24 weeks gestational age.
Have chosen an IUC (either LNG-IUC or CuT380-IUC) for contraception post abortion.
Are residents of British Columbia registered with the Medical Services Plan health care system.

Exclusion Criteria

Intention to move from BC within the next year
Intention to conceive within the next year
Uterine cavity anomalies causing distortion of the endometrial canal including fibroids of more than 5 cm, excluding repaired uterine septum
Current untreated PID, Chlamydia, gonorrhea, cervicitis or lower genital tract infection (recent infection is not a contraindication to IUC insertion[35])
Wilson's Disease (if choosing a CuT380-IUC)
Undiagnosed abnormal uterine bleeding
Known uterine or cervical malignancy or cervical dysplasia
Known or suspected progestin-dependent neoplasia, including breast cancer (if choosing a LNG-IUC)
Active liver disease or dysfunction (if choosing a LNG-IUC)
Actual benign or malignant liver tumours (if choosing a LNG-IUC)
Hypersensitivity to levonorgestrel or any of the other ingredients in the formulation or component of the container components of Mirena^® ^(if choosing a LNG-IUC)
Bacterial endocarditis
Established immunodeficiency (HIV positivity is not an exclusion unless immunodeficient)
Acute malignancies affecting blood or leukemias
Recent trophoblastic disease while hCG levels are elevated
Currently enrolled in another investigational study

Post Randomization Exclusion Criteria

Failure to undergo an abortion (ie: participants who elect to continue their index pregnancy at any time subsequent to randomization)
Uterine perforation at the time of abortion

Bleeding of more than 500 cc during abortion

Uterine cavity anomalies as outlined above

### Participating Study Clinics

All five surgical abortion clinics offering second trimester abortion in the province of BC are collaborating in this study. The geographic catchment areas for the clinics include both urban and rural areas, and service through these clinics is provided in both hospital and free-standing publicly-funded outpatient settings.

### Enrollment process

All women presenting to a participating clinic for an abortion of a pregnancy over 12 weeks and under 24 completed weeks receive information on the research study web page [35] at the time they book their appointment, and a study information brochure upon check in. Women consenting to participate complete an initial CSQ. Participants receive their chosen IUC at no cost in addition to a gift card and are randomly allocated using an internet-based randomization service [36], to immediate or planned delayed insertion, with stratification for device, parity and clinic site. This trial has been registered at controlled-trials.com (ISRCTN19506752). A pilot phase study undertook to develop, pilot test and focus group review the CSQ from an existing validated questionnaire [37], adapted for use in our population and for this study, including translation into the three most common non-English languages in our population (Cantonese, Mandarin and Punjabi). A user-friendly internet based format for each of the four languages and sampling times (intake, three, six and 12 month and annually to five years) was implemented.

### Immediate insertion group protocol

Women randomized to immediate insertion have their chosen IUC inserted by their surgeon immediately following the abortion prior to leaving the procedure or operating room. As per standard clinic protocols all women in both groups have polymerase chain reaction (PCR) testing for chlamydia and gonorrhoea prior to their abortion, and receive two grams metronidazole single observed dose as prophylaxis against postoperative infection [38]. Women deemed to be at higher risk of a sexually transmitted infection (as per criteria used by all BC clinics [38]), and those with positive PCR results, receive one gram of azithromycin as well. An ultrasound image of the IUC in situ is recorded. If an ultrasound machine is not immediately available in the operating room, arrangements are made for an ultrasound to be performed to confirm proper IUC position.

### Delayed insertion group protocol

Women randomized to planned delayed insertion are managed as closely as possible to standard practice. Participants are asked to make an appointment for follow-up and IUC insertion at 4 +/-1 weeks after the date of their abortion. All women are offered one month of an alternate contraceptive. An opportunity to return for follow-up and IUC insertion at any study clinic is available to all women, as per recommended post abortion delayed IUC insertion practice. Participants who have travelled over 100 km from a study clinic are asked to make arrangements for insertion in their home community. Study participants experience conditions as close to standard non-study delayed insertion conditions as possible. These women receive a "SmartPayment"^® ^card [39] and a prescription for the IUC. This permits participants to receive the IUC cost-free (as is the case for those able to return to the study clinic for insertion) and for the patient's pharmacy of choice to be reimbursed for the IUC. The participant is also given a requisition for a post-insertion ultrasound. The BC Medical Services Plan provides payment for the IUC insertion and the post-insertion ultrasound. This procedure is designed to as closely as possible emulate the real-life conditions each participant would experience were she not in a research study. This process eliminates possible bias in IUC insertion rate, and thus pregnancy rate, were she to take home a free study IUC or we to courier it to her designated health care provider.

### Outcome determination

CSQ are offered by mail, email or as a web-based questionnaire at 3, 6 and 12 months and annually to five years following enrollment. The CSQ collect data on expulsion of IUC, effectiveness and satisfaction with contraceptive method and insertion timing assigned, any removal of the IUC, any change to contraceptive method or intention to conceive, any interval pregnancies and their outcomes, and any adverse events. In addition participants are asked to arrange to return to any of the study sites or see their primary health care provider for a follow-up clinical examination at 3, 6 and 12 months following the abortion. Information from clinical visits and CSQ will be used in conjunction with BC administrative health database information to determine outcomes.

### Ethical Aspects

This study has received institutional review board approval from the following research ethics boards: the University of British Columbia-Children's and Women's Research Ethics Board (H10-00306), the Interior Health Authority Research Ethics Board (2010-028) and the Vancouver Island Health Authority Clinical Research Ethics Board (C2010-47). A Data and Safety Monitoring Board has been established consisting of an Obstetrician-Gynaecologist, a biostatistician, an expert in Population and Public Health and database linkage research, and an economist specializing in population health pharmaceutical economics, all from Canadian universities outside of BC, and each being independent of all members of the research team. Funding for this study is primarily through grants from the Canadian Institutes for Health Research, with pilot funding and administrative support from the Women's Health Research Institute, and the University of British Columbia. Donation of 385 free Mirena devices was provided by Bayer Inc. as their sole contribution to the research study.

### Analysis

*Primary outcome *will be examined in an intention-to-treat framework as pregnancy rate at one year among women randomized to immediate insertion compared to women randomized to a planned insertion at 4 weeks ("delayed insertion") for each of the two IUC devices. For example, if women in the delayed group present for insertion after the specified 4+/-1 week insertion window, or even at the time of a subsequent abortion or delivery, we will insert the device at the women's request and her assignment for the primary analysis will not change. Similarly if for any reason a woman assigned to the immediate insertion group is unable to have her insertion immediately (for example, should she have an unattended expulsion/delivery of her pregnancy prior to the planned surgical abortion) then insertion will be offered at the time the woman and her physician would normally undertake to do so, and whether or not her insertion ever occurs, her assignment for the purpose of analysis will not change. Thus our primary outcome of pregnancy rate at one year reflects real life conditions.

*Secondary outcomes *include: costs and cost effectiveness, cumulative annual pregnancy rate, device insertion rate, loss to follow-up; continuation of method; adverse events (such as infection or perforation); expulsion; outcomes among those participants who were chlamydia positive at the time of insertion; satisfaction with IUC chosen and with insertion timing assigned. These outcomes will be assessed initially at one year, then annually through the five year device effectiveness period.

### Operational definition for outcome

Although our outcome of one year pregnancy rate is conceptually simple, exact determination of conception dates is clearly infeasible. Consequently our actual outcome definition provides a pragmatic approximation based on the varying exactitude of imputations based on provincial Medical Service Plan billings related to abortions, miscarriages, still births and live births. Subsequent abortions performed within our study clinics for enrolled participants will be noted along with specific clinical information on pregnancy duration. For abortions performed elsewhere such as those for BC university students studying out of province, or those performed by individual physicians at rural or remote hospitals within BC, exact gestation may not be available. In the BC health administrative databases abortions are billed as under 7 weeks for medical abortions, and as under 14 weeks, 14 weeks to under 18 weeks, and 18 weeks and over for surgical abortions. Miscarriages by definition occur anytime under 20 weeks or are classed as still birth when over 20 weeks of gestation. Thus for subsequent pregnancies where the specific gestation is unknown, we will consider a pregnancy to have occurred in the first year according to the following conservative adjustments to one year follow-up dates. (see Table 3.)

**Table 3:** Operational definition of a pregnancy as derived from administrative billing data

Billing Type	Nominal weeks of gestation	Cut-off for operational definition
Abortion (medical)	< 7 weeks*	1 year + 4 weeks
Abortion (surgical)	< 14 weeks	1 year + 6 weeks
Abortion (surgical)	14-18 weeks	1 year + 13 weeks
Abortion (surgical)	≥ 18 weeks	1 year + 17 weeks
Miscarriage	< 20 weeks	1 year + 11 weeks
Still birth	≥ 20 weeks	1 year + 25 weeks
Live birth	Birth date - GA	1 year + XX*

*In Canada, mifepristone is not available. Medication-induced abortions using methotrexate and misoprostol are offered in British Columbia up to a maximum of 49 days (7 weeks) from last menstrual period.

** Where XX = median conceptual age for live births in BC

### Analysis methods

We will compare one year pregnancy rates using a chi-squared test declaring significance if p < .05 and provide 95% confidence intervals for the difference in pregnancy rates using the large sample normal approximation for differences in proportions. This simple approach is valid so long as no systematic difference in follow-up occurs between groups. As a check, we will also conduct analysis to account for partial follow-up. Since our outcome definition is essentially composite and the relevant risk periods differ by components, Kaplan-Meier estimates for each component event will be determined and composite estimates will be obtained by summing the estimated cumulative event rates (calculated as 1 - the survival function) at the time-points indicated in our operational definitions. Confidence intervals around the difference in these estimates will be calculated using the bootstrap.

Rates for all secondary outcomes will be calculated for events occurring within one year following abortion, and annually for five years, using the follow-up questionnaires, direct access to clinical follow up visit records, and billing and procedure coding data from the administrative health system databases. Multivariate logistic regression will be used to examine demographic, socioeconomic, and obstetrical factors in relationship to primary and secondary objectives.

### Survey Analysis

The quantitative data from the CSQ will be analyzed using descriptive statistics. The CSQ contain several scales providing composite scores that can be used to indicate differences in the secondary outcomes. Open ended questions will be analyzed through content analysis focusing on key topics.

### Health Economic Analysis

Economic analysis seeks to provide comparative information on the costs and benefits of alternative clinical strategies. In this study the strategy of interest is the immediate IUC insertion after second trimester abortion versus the delayed insertion. The economic analysis will provide estimates of the improvements in benefits associated with such a potential health policy change, and the associated costs.

Given the nature of the clinical condition, it would be inappropriate for benefits to be measured using quality-adjusted life years (QALYs). Therefore a cost-effectiveness analysis will be conducted whereby the measure of effect is simply the number of unintended pregnancies prevented. The primary focus for the economic analysis will therefore be estimating resource use and costs, with the main measure of benefit not valued explicitly within the analysis but a presumption that avoiding unintended pregnancies is inherently a positive outcome.

A broad perspective will be adopted to include costs incurred within the health care sector (such as contraception, abortion, medications etc.), those incurred by other sectors (e.g. social services for fostering and adoption) and those incurred by women and their families. In line with the main trial, the time span for collection of resource and cost data will be one year in the first instance and then data collection will be extended to five years. The process of collecting resource use data will be undertaken separately from data collection on unit costs. Data on use of resources in the health and welfare sectors will be gathered from the linked health and administrative databases of the government of British Columbia and managed by Population Data BC. Similarly, data on use of resources in other sectors is also available from routine sources, with linking of data to be performed for this work. Unit costs will be obtained and attached to resource items in order that a cost can be calculated for each trial participant. Unit costs will be obtained from published sources and centres participating in the trial.

A within-trial economic analysis will be carried out, adopting an incremental approach in that data collection will concentrate on resource use and outcome differences between trial arms. As the majority of cost data are skewed, and the mean cost of each procedure is of importance, non-parametric bootstrapping will be used to estimate confidence intervals around the mean costs and benefits. To reflect the differential timing of costs being incurred and benefits being experienced, discounting will be applied according to standard guidelines.

The results will be presented as incremental cost-effectiveness ratios (ICERs) and cost-effectiveness acceptability curves (CEACs). The robustness of the results will be explored using sensitivity analysis, to investigate uncertainties in the data, the analysis methods and the generalizability of the results to other settings.

## Discussion

### Anticipated Limitations

#### Changes in intention-to-conceive

Due to our exclusion criteria, we recruit only women who do not intend to conceive within the first year after enrollment. Nevertheless, in this study population with an anticipated mean age of 24, we fully anticipate some individuals will change their intent to conceive over the first and subsequent study years. We will account for this in two ways. First we ask at each CSQ (three, six and 12 months and annually to 5 years) about the intent to conceive, and second, we assume randomization will distribute those who have intended pregnancies within the study period evenly to both arms of the study.

#### Expulsions

Most women are aware of an IUC expulsion [40], and should it occur, will make arrangements for alternate contraception. This alternate contraception may be a replacement device or a change of contraceptive method. Each participant will be provided with a toll free number enabling them to contact the Principal Investigator (WVN) at any time. In addition, the study team will monitor follow up visits, CSQ, Medical Services Plan billings and prescription records of alternate contraception prescribed or an IUC inserted. In this manner, we believe we will be able to estimate device expulsion rate with a fair degree of accuracy. In order to most accurately reflect usual contraceptive conditions in the event of an expulsion, we are not providing a free replacement device. We have stratified at randomization for parity, as this may be a factor in expulsion.

### Summary

This paper highlights considerations of design, implementation and evaluation of a randomized controlled trial expected to provide rigorous evidence for appropriate insertion timing and health economic considerations for the two most common forms of intrauterine contraception after second trimester abortion.

## Abbreviations

BC: British Columbia, a province in Canada; CEACs: Cost-Effectiveness Acceptability Curves; CSQ: Contraception Satisfaction Questionnaires; CuT380-IUC: Copper Intrauterine Contraception ("Flexi-T380(+)^®^" Prosan, The Netherlands); ICERs: Incremental Cost-Effectiveness Ratios; IUC: Intrauterine Contraception; LNG-IUC: Levonorgestrel-releasing IUC ("Mirena^®^", Bayer Inc, Canada); PCR: Polymerase Chain Reaction; QALYs: Quality-Adjusted Life Years; RCT Randomized Controlled Trial

## Competing interests

The authors declare that they have no competing interests.

## Authors' contributions

WN, JK, JS, RB, SB, LD made substantial contributions to conception and design of this study. WN, KT, LD contributed to acquisition of data. All authors contributed to analysis and interpretation of data; WN, JK, JS, RB, SB drafted the protocol and protocol manuscript; and all authors contributed to revising it critically for important intellectual content and have read and approved the final manuscript.

## Authors' information

The authors work within the Contraception & Abortion Research Team of the University of British Columbia. This team is supported by the Women's Health Research Institute of the Provincial Health Services Authority of British Columbia, Canada. WVN is supported by the Clinician Scholar's Program within the UBC Faculty of Medicine.
